# Identification of prognostic lipid droplet-associated genes in pancreatic cancer patients via bioinformatics analysis

**DOI:** 10.1186/s12944-021-01476-y

**Published:** 2021-06-02

**Authors:** Rubing Bai, Artur Rebelo, Jörg Kleeff, Yoshiaki Sunami

**Affiliations:** Department of Visceral, Vascular and Endocrine Surgery, University Medical Center, Martin-Luther-University Halle-Wittenberg, Ernst-Grube-Strasse 40, 06120 Halle (Saale), Germany

**Keywords:** Bioinformatics, GEPIA, Lipid droplet-associated genes, Pancreatic cancer, Lipid metabolism

## Abstract

**Background:**

Pancreatic cancer is the fourth leading cause of cancer deaths in the United States both in females and in males, and is projected to become the second deadliest cancer by 2030. The overall 5-year survival rate remains at around 10%. Cancer metabolism and specifically lipid metabolism plays an important role in pancreatic cancer progression and metastasis. Lipid droplets can not only store and transfer lipids, but also act as molecular messengers, and signaling factors. As lipid droplets are implicated in reprogramming tumor cell metabolism and in invasion and migration of pancreatic cancer cells, we aimed to identify lipid droplet-associated genes as prognostic markers in pancreatic cancer.

**Methods:**

We performed a literature search on review articles related to lipid droplet-associated proteins. To select relevant lipid droplet-associated factors, bioinformatics analysis on the GEPIA platform (data are publicly available) was carried out for selected genes to identify differential expression in pancreatic cancer versus healthy pancreatic tissues. Differentially expressed genes were further analyzed regarding overall survival of pancreatic cancer patients.

**Results:**

65 factors were identified as lipid droplet-associated factors. Bioinformatics analysis of 179 pancreatic cancer samples and 171 normal pancreatic tissue samples on the GEPIA platform identified 39 deferentially expressed genes in pancreatic cancer with 36 up-regulated genes (*ACSL3*, *ACSL4*, *AGPAT2*, *BSCL2*, *CAV1*, *CAV2*, *CAVIN1*, *CES1*, *CIDEC*, *DGAT1*, *DGAT2*, *FAF2*, *G0S2*, *HILPDA*, *HSD17B11*, *ICE2*, *LDAH*, *LIPE*, *LPCAT1*, *LPCAT2*, *LPIN1*, *MGLL*, *NAPA*, *NCEH1*, *PCYT1A*, *PLIN2, PLIN3, RAB5A*, *RAB7A*, *RAB8A*, *RAB18*, *SNAP23*, *SQLE*, *VAPA*, *VCP*, *VMP1*) and 3 down-regulated genes (*FITM1*, *PLIN4*, *PLIN5*). Among 39 differentially expressed factors, seven up-regulated genes (*CAV2*, *CIDEC*, *HILPDA*, *HSD17B11*, *NCEH1*, *RAB5A*, and *SQLE*) and two down-regulation genes (*BSCL2* and *FITM1*) were significantly associated with overall survival of pancreatic cancer patients. Multivariate Cox regression analysis identified CAV2 as the only independent prognostic factor.

**Conclusions:**

Through bioinformatics analysis, we identified nine prognostic relevant differentially expressed genes highlighting the role of lipid droplet-associated factors in pancreatic cancer.

## Background

Pancreatic cancer is the fourth leading cause of cancer deaths in the United States both in females and males [1], and is predicted to be the second most common cancer by 2030 [2]. Although recent therapeutic advances such as more effective adjuvant and neo-adjuvant chemotherapies, together with more radical and safer surgery, pancreatic cancer prognosis is very poor and the overall 5-year survival rate is still 10% [1, 3]. Metabolic reprogramming has been recognized as a hallmark of cancer [4]. Aberrant lipid synthesis and reprogrammed lipid metabolism are both associated with the development and progression of pancreatic cancer [5]. This can be because lipids such as phospholipid bilayers are fundamental structural components enabling cellular proliferation [5]. Lipid droplets (LDs) are ubiquitous intracellular organelles that store neutral lipids, such as, triacylglycerides, and cholesterol esters [6, 7]. The storage of neutral lipids in LDs is important for protecting cells from lipotoxicity due to the buildup of excess lipids in cell membranes [7]. Some cancer cells accumulate massive amount of LDs [7]. LDs are further implicated to mediate the proliferation, invasion, metastasis, as well as chemotherapy resistance in several types of cancer [8]. Oncogenic KRAS, which is the most important driver for pancreatic cancer development, controls the storage and utilization of LD supporting reprogramming of tumor cell metabolism, invasion and migration [9]. LDs are composed of a monolayer of phospholipids together with a variety of proteins such as structural proteins, membrane transport proteins, and enzymes [8]. LDs can associate with most other cellular organelles through membrane contact sites mediated by a set of proteins [10]. Since LD-associated proteins play an important role in dynamics of LD, we hypothesized that LD-associated factors may be associated with the outcome of pancreatic cancer patients.

## Methods

### Literature search for selecting lipid droplet-associated factors

To identify lipid droplet-associated factors, we performed a literature search in the PubMed database related to lipid droplet-associated proteins (last search date: July 2020). All publications with keywords “lipid droplet-associated protein” and the category “review” were collected. There was no restriction on the publication period. We analyzed all retrieved articles [11–39], and selected factors that were described to localize on lipid droplet including factors on contact sites to other organelles, such as endoplasmic reticulum and mitochondria.

### Gene expression profiling interactive analysis (GEPIA) bioinformatics analysis

For gene expression profiling and overall survival analysis, we conducted bioinformatics analysis on the GEPIA platform (http://gepia.cancer-pku.cn/) [40]. GEPIA is an online analysis tool for processing high-throughput RNA sequencing expression data of bulk tumorous and normal samples based on the Cancer Genome Atlas (TCGA) (https://portal.gdc.cancer.gov/) and the Genotype-Tissue Expression (GTEx, https://www.gtexportal.org/) databases. Our analysis included 179 pancreatic cancer samples and 171 normal pancreatic tissue samples. Dot maps of selected genes were generated. Furthermore, the GEPIA differential analysis module was used for analyzing gene expression profiles of pancreatic cancer and paired normal samples, and for screening differentially expressed genes (DEGs) between tumor and normal tissues. DEGs were further analyzed for overall survival (OS) on the GEPIA platform. Median expression was used as the threshold between high expression and low expression cohorts. The Human Protein Atlas version 20.1 (https://www.proteinatlas.org) [41] was used to analyze DEGs at the protein level by immunohistochemistry.

### Statistical analysis

Statistical analysis was performed through the plug-in units of GEPIA. Analysis of variance (ANOVA) and Limma package plug-in were performed for screening DEGs. The data analysis function of Limma package is for the construction of linear models and differential expression for RNA-seq data. Genes with log2 (fold change) > 1 or < − 1, and a *P* value < 0.05 were considered DEGs. *P*-values were calculated as false discovery rate (FDR)-adjusted *P*-values with the Limma packages. OS analysis was performed using the Kaplan-Meier survival plots tool in the GEPIA platform. Via Log-rank test, log-rank *P*-values, hazards ratios (HR), and Cox *P*-values were obtained. Univariate and multivariate Cox regression analysis was carried out via Cox Proportional-Hazards (CoxPH) function in R. *P* < 0.05 was considered statistically significant.

## Results

### Selection of lipid droplet-associated factors for the bioinformatics analysis

Following the literature search and analysis, 65 factors were retrieved as LD-associated factors. The selecting criteria included factors not only directly implicated as LD factors, but also factors localized on contact sites of LD with other organelles such as endoplasmic reticulum and mitochondria. The majority of publications indicated perilipin family members PLIN1–5 (Table 1). The selected factors further included: Abhydrolase 5 (ABHD5, also known as Comparative gene identification CGI-58), Acetyl-CoA acetyltransferase 1 (ACAT1), Acyl-CoA synthetase long chain family member (ACSL) 3–4, 1-acylglycerol-3-phosphate O-acyltransferase 2 (AGPAT2, also known as Berardinelli-Seip congenital lipodystrophy BSCL1), Angiopoietin like 8 (ANGPTL8, also known as C19orf80), Apolipoprotein APOA4, APOB, Ancient ubiquitous protein 1 (AUP1), BSCL2 (Seipin), Caveolin 1 (CAV1, also known as BSCL3), CAV2, Caveolae-associated protein 1 (CAVIN1), Carboxyesterase 1 (CES1), Cell death-inducing DNA fragmentation factor-like effector (Cide) family members (CIDEA, CIDEB, CIDEC), Diacylglycerol O-Acyltransferase (DGAT) 1–2, Fas-associated factor 2 (FAF2, also known as UBX domain UBXD8), Fat storage inducing transmembrane protein (FITM) 1–2, G0/G1 switch protein (G0S2), Glycerol-3-phosphate acyltransferase 4 (GPAT4), Hypoxia inducible LD associated (HILPDA), Hydroxysteroid HSD17B11, HSD17B13, Interactor of little elongation complex ELL ICE2, LD-associated hydrolase (LDAH), Lipase E (LIPE, also known as Hormone sensitive lipase HSL), Lysophosphatidylcholine Acyltransferase (LPCAT) 1–2, phosphatidic acid phosphohydrolase Lipin 1 (LPIN1, PAP1), Lanosterol synthase (LSS), Methyltransferase like 7A (METTL7A, also known as AAMB), METTL7B (also known as Associated with LD protein I, ALDI), Monoglyceride lipase (MGLL), Microsomal triglyceride transfer protein (MTTP), *N*-ethylmaleimide-sensitive factor (NSF)-attachment protein α (NAPA, also known as α-SNAP), Neutral cholesterol ester hydrolase 1 (NCEH1), NSF, Oxysterol binding protein like 2 (OSBPL2, also known as ORP2), Phosphate Cytidylyl transferase PCYT1A (also known as CTP:phosphocholine cytidyltransferase CCTA), Phosphatidylethanolamine *N*-methytransferase (PEMT), Phospholipase 1 (PLD1), Patatin-like phospholipase domain containing 2 (PNPLA2), PNPLA3, PNPLA5, RAS oncogene family RAB5A, RAB7A, RAB8A, RAB18, SNAP23, Squalene epoxidase (SQLE), Synthaxin 5 (STX5), Ubiquitin conjugating enzyme UBE2G2, Vesicle associated membrane protein 4 (VAMP4), Vesicle-associated membrane protein-associated protein (VAPA), Valosin containing protein (VCP, also known as p97), and Vesicular membrane protein 1 (VMP1) (Table 1).

**Table 1:** Lipid droplet-associated factors retrieved by literature search. Sixty-five factors were identified as lipid droplet-associated factors by literature search and analysis.

Factor name	References	Factor name	References	Factor name	References
ABHD5 (CGI-58)	[11, 12, 15, 17–20, 22–29, 34, 35, 37, 38]	ACAT1	[28, 33]	ACSL3	[14, 15, 18, 35]
ACSL4	[18, 35]	AGPAT2 (BSCL1)	[18, 21]	ANGPTL8 (C19orf80)	[21]
APOA4	[18]	APOB	[18]	AUP1	[15, 18]
BSCL2 (Seipin)	[14, 18, 20]	CAV1 (BSCL3)	[18, 20, 29]	CAV2	[18, 29]
CAVIN1	[18]	CES1	[18, 32]	CIDEA	[13, 18–20, 22–24, 28–31, 39]
CIDEB	[13, 18, 19, 22–24, 31, 39]	CIDEC (FSP27)	[13, 17–20, 22–25, 28–31, 39]	DGAT1	[11, 15, 18, 33, 38]
DGAT2	[11, 14, 15, 18, 20, 21, 33, 35, 38]	FAF2 (UBXD8)	[15, 18]	FITM1	[19]
FITM2	[14, 19, 20]	G0S2	[11, 17, 19, 20, 25, 28]	GPAT4	[15, 18, 21, 38]
HILPDA	[11, 17, 19]	HSD17B11	[15, 18, 35]	HSD17B13	[15, 18]
ICE2	[14]	LDAH	[18]	LIPE (HSL)	[11, 15, 17, 18, 20, 22, 23, 28, 29, 31, 32, 34, 35, 37, 38]
LPCAT1	[18, 21]	LPCAT2	[18, 21]	LPIN1	[38]
LSS	[35]	METTL7A (AAMB)	[15]	METTL7B (ALDI)	[15]
MGLL	[11]	MTTP	[20]	NAPA	[38]
NCEH1	[18]	NSF	[38]	OSBPL2 (ORP2)	[21]
PCYT1A (CCTA)	[18, 20]	PEMT	[21]	PLD1	[38]
PLIN1	[11, 14–20, 22–25, 28–33, 35, 36, 38]	PLIN2 (ADRP)	[11, 14–20, 22–25, 28, 30–33, 35, 36, 38]	PLIN3 (TIP47)	[11, 14, 16–20, 22–25, 28, 31–36, 38]
PLIN4 (S3–12)	[11, 14, 16–20, 22–24, 28, 33, 35]	PLIN5 (OXPAT/MLDP/LSDP5)	[11, 14, 16–20, 22–25, 28, 31, 33–35]	PNPLA2 (ATGL)	[11, 12, 15, 17–25, 28, 29, 31–38]
PNPLA3 (ADPN)	[12, 18, 27]	PNPLA5	[21]	RAB5A	[18, 36, 38]
RAB7A	[21, 36]	RAB8A	[18]	RAB18	[14, 15, 18, 20, 24, 38]
SNAP23	[38]	SQLE	[35]	STX5	[38]
UBE2G2	[15]	VAMP4	[38]	VAPA	[21]
VCP (p97)	[15, 18]	VMP1	[14]		

### Expression of a large number of lipid droplet-associated genes is significantly altered in pancreatic cancer patient specimens

**Fig. 1:**
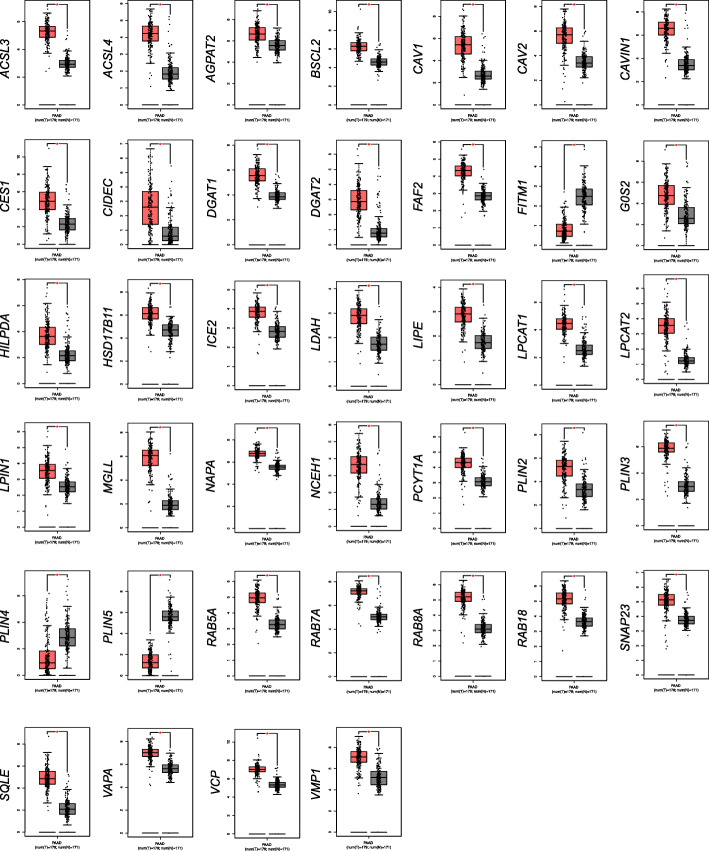
The mRNA expression levels of 39 differentially expressed genes in pancreatic cancer. Gene mRNA expression level of lipid droplet-associated factors was analyzed with the GEPIA platform. The 39 differentially expressed genes (*ACSL3*, *ACSL4*, *AGPAT2*, *BSCL2*, *CAV1*, *CAV2*, *CAVIN1*, *CES1*, *CIDEC*, *DGAT1*, *DGAT2*, *FAF2*, *FITM1*, *G0S2*, *HILPDA*, *HSD17B11*, *ICE2*, *LDAH*, *LIPE*, *LPCAT1*, *LPCAT2*, *LPIN1*, *MGLL*, *NAPA*, *NCEH1*, *PCYT1A*, *PLIN2, PLIN3, PLIN4*, *PLIN5, RAB5A*, *RAB7A*, *RAB8A*, *RAB18*, *SNAP23*, *SQLE*, *VAPA*, *VCP*, *VMP1*) were depicted via bar plots between pancreatic cancer versus pancreas normal tissue (T: pancreatic cancer tissue, N: pancreas normal tissue)

We analyzed the RNA-seq expression data of 65 genes related to lipid metabolism in 179 PDAC and 171 normal samples. Thirty-nine differentially expressed genes (DEGs), with 36 up-regulated (*ACSL3*, *ACSL4*, *AGPAT2*, *BSCL2*, *CAV1*, *CAV2*, *CAVIN1*, *CES1*, *CIDEC*, *DGAT1*, *DGAT2*, *FAF2*, *G0S2*, *HILPDA*, *HSD17B11*, *ICE2*, *LDAH*, *LIPE*, *LPCAT1*, *LPCAT2*, *LPIN1*, *MGLL*, *NAPA*, *NCEH1*, *PCYT1A*, *PLIN3*, *RAB5A*, *RAB7A*, *RAB8A*, *RAB18*, *SNAP23*, *SQLE*, *VAPA*, *VCP*, *VMP1*) and 3 down-regulated genes (*FITM1*, *PLIN4*, *PLIN5*) were identified in bulk pancreatic cancer as compared to normal pancreatic tissues (Fig. 1). Statistical data are summarized in Table 2.

**Table 2:** Statistical data of lipid droplet-associated factors identified as DEGs. Differential expression analysis of lipid droplet-associated genes was performed on the GEPIA platform. The statistical data of 39 DEGs between pancreatic cancer versus normal pancreatic tissues are shown

Gene Symbol	Gene ID	Median (Tumor)	Median (Normal)	Log2(Fold Change)	Adj. *P*-value
ACSL3	ENSG00000123983.13	38.869	6.610	2.389	9.39e-76
ACSL4	ENSG00000068366.19	17.629	2.540	2.396	4.33e-66
AGPAT2	ENSG00000169692.12	100.071	46.240	1.097	6.24e-17
BSCL2	ENSG00000168000.14	77.772	23.070	1.710	9.80e-44
CAV1	ENSG00000105974.11	42.261	5.060	2.836	6.07e-52
CAV2	ENSG00000105971.14	51.282	9.600	2.302	2.89e-39
CAVIN1	ENSG00000177469.12	95.510	9.460	3.879	1.89e-40
CES1	ENSG00000198848.12	28.929	3.970	2.590	4.39e-31
CIDEC	ENSG00000187288.10	5.060	0.490	2.024	1.74e-20
DGAT1	ENSG00000185000.9	45.730	13.480	1.690	3.74e-42
DGAT2	ENSG00000062282.14	6.230	0.710	2.080	5.63e-36
FAF2	ENSG00000113194.12	19.030	6.270	1.462	1.49e-59
FITM1	ENSG00000139914.6	0.640	4.620	−1.777	7.21e-56
G0S2	ENSG00000123689.5	25.751	5.000	2.157	9.55e-22
HILPDA	ENSG00000135245.9	11.370	3.420	1.485	8.02e-28
HSD17B11	ENSG00000198189.10	67.930	24.891	1.413	7.59e-32
ICE2	ENSG00000128915.11	13.590	6.080	1.043	5.07e-38
LDAH	ENSG00000118961.14	6.480	2.310	1.176	2.26e-45
LIPE	ENSG00000079435.9	5.960	1.870	1.278	2.58e-21
LPCAT1	ENSG00000153395.9	21.251	4.670	1.972	4.51e-53
LPCAT2	ENSG00000087253.11	10.760	1.330	2.335	5.24e-57
LPIN1	ENSG00000134324.11	10.770	4.790	1.024	1.04e-23
MGLL	ENSG00000074416.13	65.639	2.700	4.171	6.12e-80
NAPA	ENSG00000105402.7	108.774	45.581	1.237	7.56e-36
NCEH1	ENSG00000144959.9	11.570	1.480	2.342	3.75e-57
PCYT1A	ENSG00000161217.11	18.930	7.370	1.252	9.92e-42
PLIN2	ENSG00000147872.9	37.980	8.970	1.967	2.64e-29
PLIN3	ENSG00000105355.8	58.351	6.890	2.911	1.13e-74
PLIN4	ENSG00000167676.4	0.900	6.180	−1.918	5.30e-13
PLIN5	ENSG00000214456.8	1.340	46.610	−4.347	3.06e-86
RAB5A	ENSG00000144566.10	30.130	8.570	1.702	1.41e-58
RAB7A	ENSG00000075785.12	148.024	31.851	2.182	1.25e-66
RAB8A	ENSG00000167461.11	35.579	7.510	2.104	3.17e-70
RAB18	ENSG00000099246.16	34.750	11.450	1.522	4.92e-49
SNAP23	ENSG00000092531.9	33.981	12.280	1.397	1.09e-34
SQLE	ENSG00000104549.11	28.019	3.220	2.782	4.34e-62
VAPA	ENSG00000101558.13	128.952	48.600	1.390	1.38e-39
VCP	ENSG00000165280.15	128.470	39.180	1.688	1.08e-50
VMP1	ENSG00000062716.10	136.901	34.789	1.946	7.73e-33

### Lipid droplet-associated gene expression of *CAV2*, *CIDEC*, *HILPDA*, *HSD17B11*, *NCEH1*, *RAB5A*, and *SQLE* is associated with poor survival while expression of *BSCL2* and *FITM1* is associated with longer overall survival of pancreatic cancer patients

**Fig. 2:**
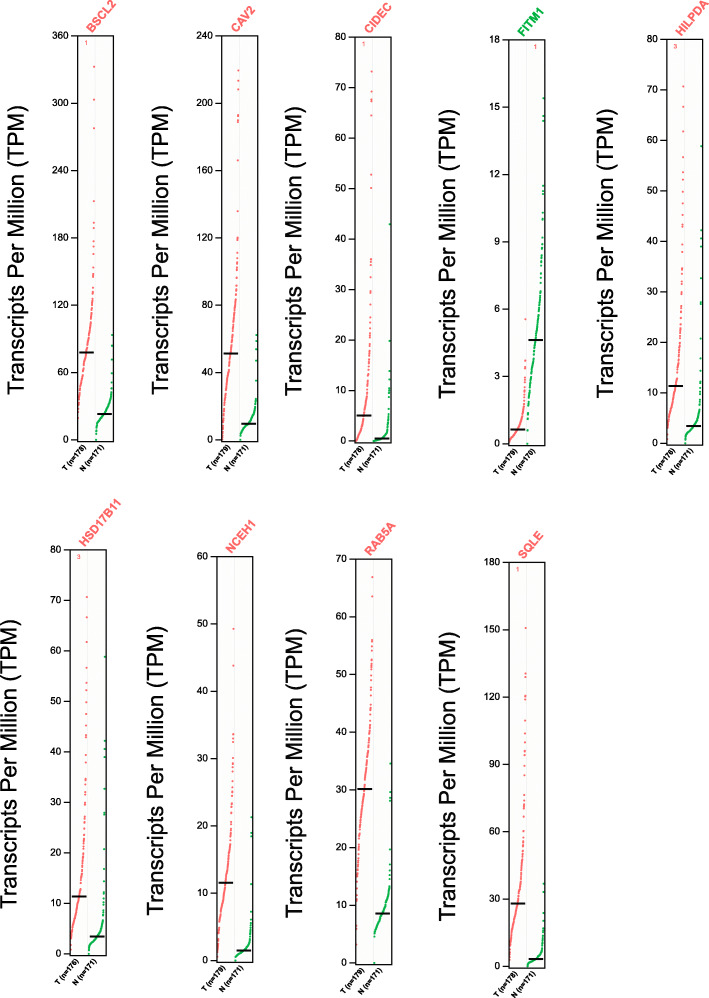
Prognostic gene expression profiling. The dot plots profiling of 9 prognostic gene expression (*BSCL2, CAV2*, *CIDEC*, *FITM1, HILPDA*, *HSD17B11*, *NCEH1*, *RAB5A*, and *SQLE*) were generated across pancreatic cancer and paired pancreas samples. Each dot represents an independent cancer or normal samples

**Fig. 3:**
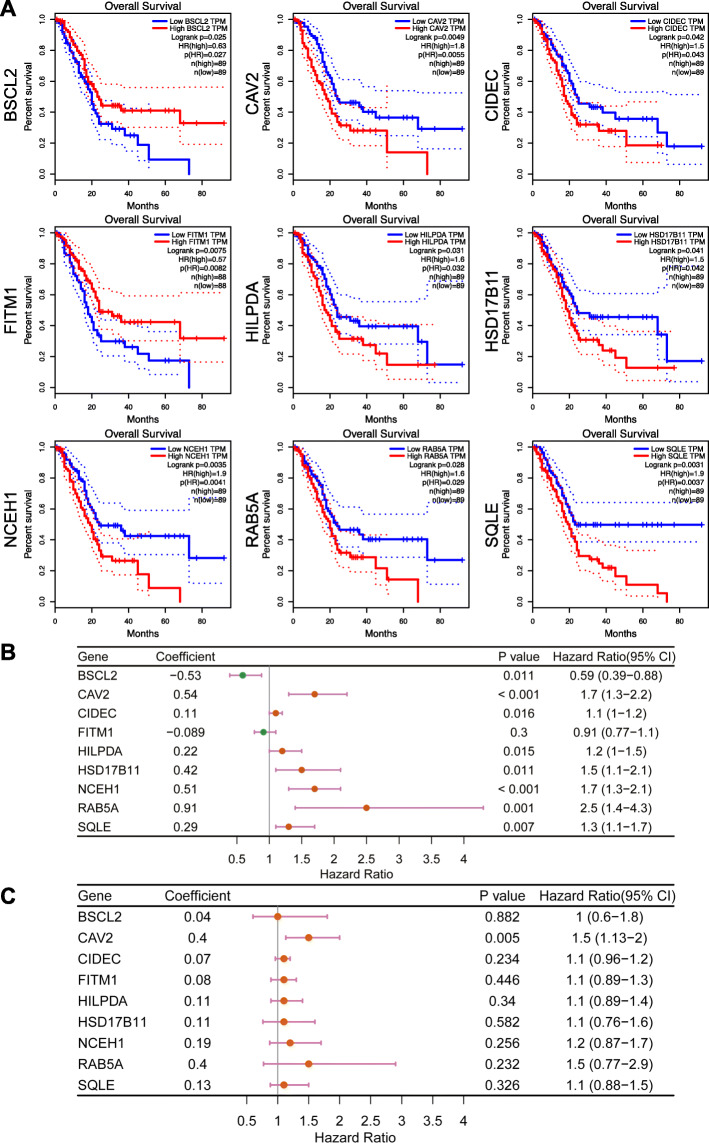
Analysis of 9 genes at pancreatic cancer overall survival. **a**. Overall survival analyses of 9 prognostic genes (*BSCL2, CAV2*, *CIDEC*, *FITM1, HILPDA*, *HSD17B11*, *NCEH1*, *RAB5A*, and *SQLE*) at pancreatic cancer based on the GEPIA database. **b**. Univariate Cox regression analysis of nine prognostic genes. **c**. Multivariate Cox regression analysis of nine prognostic genes

**Fig. 4:**
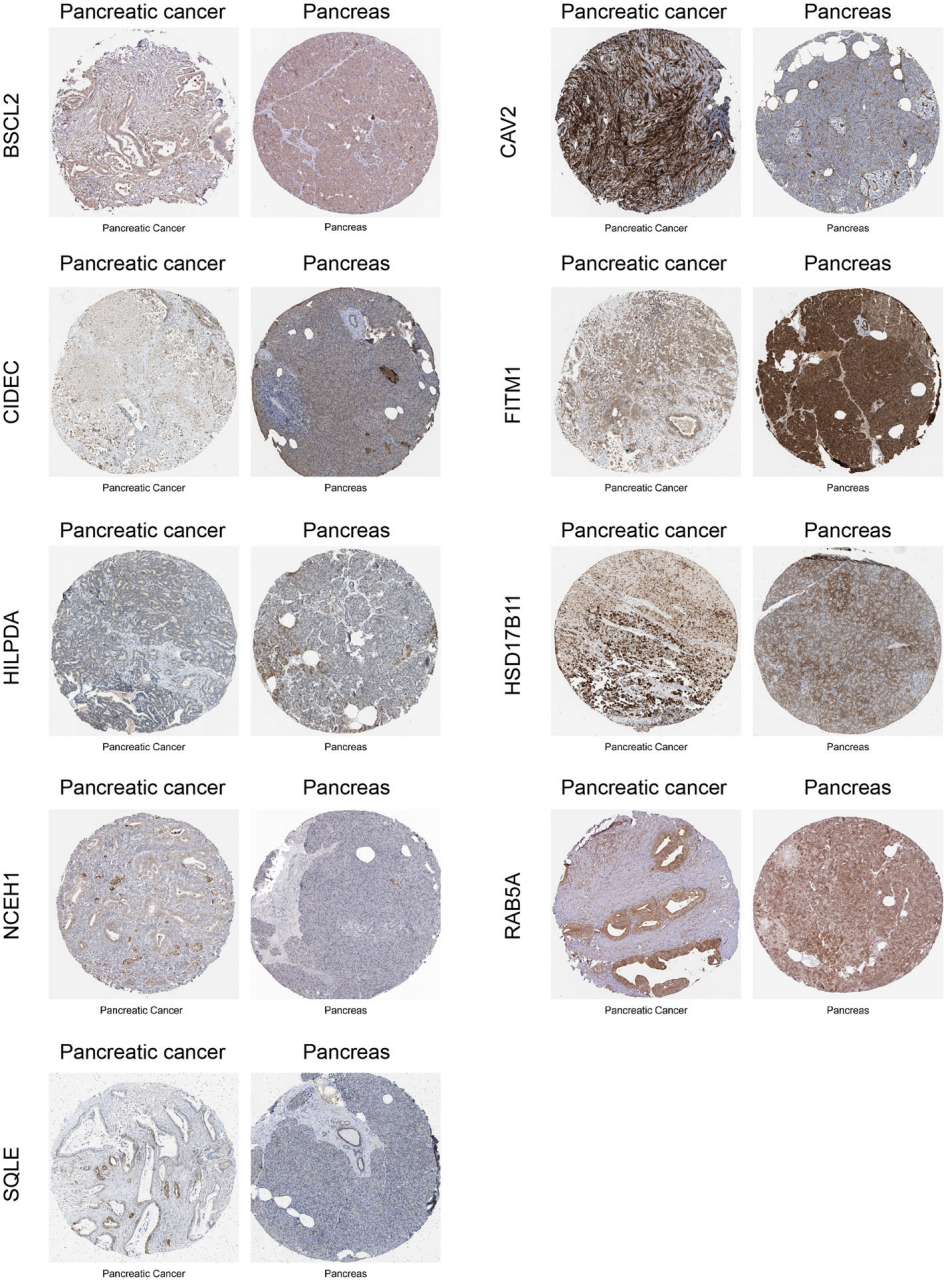
Representative immunohistochemistry of nine lipid droplet-associated genes between pancreatic cancer and normal pancreas tissues in the Human Protein Atlas database

Next, overall survival (OS) analysis of 39 DEGs was performed on the GEPIA platform. The results revealed that up-regulation of seven genes (*CAV2*, *CIDEC*, *HILPDA*, *HSD17B11*, *NCEH1*, *RAB5A*, and *SQLE*) and down-regulation of *FITM1* were significantly associated with a decrease in OS. Interestingly, up-regulation of *BSCL2* was associated with favorable OS. Hence, through the above analysis, nine prognostic DEGs of lipid droplet-associated factors were identified in pancreatic cancer (Fig. 2, 3a). To further verify, whether these nine genes have prognostic power, we performed univariate Cox proportional hazards regression analysis and calculated hazard ratios (HRs) and 95% confidence intervals (CIs). Among nine prognostic DEGs, *FITM1* was not a significant prognostic factor in the univariate Cox regression analysis (coefficient: -0.089, HR: 0.91 (0.77–1.1), *P* = 0.3), whereas the other eight genes were confirmed as prognostic factors (*BSCL2* coefficient: -0.53, HR: 0.59 (0.39–0.88), *P* = 0.011; *CAV2* coefficient: 0.54, HR: 1.7 (1.3–2.2), *P* < 0.001; *CIDEC* coefficient: 0.11, HR: 1.1 (1–1.2), *P* = 0.016; *HILPDA* coefficient: 0.22, HR: 1.2 (1–1.5), *P* = 0.015; *HSD17B11* coefficient: 0.42, HR: 1.5 (1.1–2.1), *P* = 0.011; *NCEH1* coefficient: 0.51, HR: 1.7 (1.3–2.1), *P* < 0.001; *RAB5A* coefficient: 0.91, HR: 2.5 (1.4–4.3), *P* = 0.001; *SQLE* coefficient: 0.29, HR: 1.3 (1.1–1.7), *P* = 0.007) (Fig. 3b). To identify, whether these genes were independent prognostic factors, we performed multivariate Cox proportional hazards regression analysis. Among the prognostic DEGs, only *CAV2* was an independent prognostic factor (coefficient: 0.4, HR: 1.5, *P* = 0.005) (Fig. 3c). We further analyzed protein expression of the nine DEGs on the Human Protein Atlas database. Representative immunohistochemistry pictures of the DEGs are shown in Fig. 4.

Taken together, LD-associated factors seem to be relevant in pancreatic cancer since there are linked by bioinformatics analyses to overall survival of pancreatic cancer patients.

## Discussion

LD accumulation in non-adipose tissues has been recognized as a new hallmark of cancer cells [8]. Higher LD content has been reported in colorectal, breast, prostate cancer, hepatocellular carcinoma, renal cell carcinoma and glioblastoma [8]. LDs have been implicated to mediate aspects of proliferation, invasion, metastasis, as well as chemotherapy resistance in several types of cancer [8]. Increased storage of lipids in LDs has been suggested to be beneficial for the survival of cancer cells. Increased LD contents may expand the energy source for the metabolic need of proliferative cancer cells [8]. Storage of excess FAs and cholesterol in LDs can prevent lipotoxicity and endoplasmic reticulum (ER) stress [8]. Crucial regulators in LD homeostasis include structural proteins, membrane transport proteins and enzymes [8]. We hypothesized that expression of LD-associated factors is relevant in pancreatic cancer, because LD-associated factors support the storage of neutral lipids in lipid droplets providing energy source for cancer cells and potentially protecting cancer cells from lipotoxicity. Furthermore, LDs can associate with most other cellular organelles such as ER, nucleus, mitochondria, peroxisomes, and lysosomes through membrane contact sites [10]. It is not known whether LDs can be transferred between cells, or can be secreted into the blood stream. It has been shown that tumors signal over long distances to sites of future metastases to promote formation of a pre-metastatic niche that potentially supports growth of disseminated tumor cells upon their arrival [42]. If lipid droplets act as “transporters” of lipids and signaling molecules between cells, LDs and LD-associated factors might be involved in metastasis. The molecular mechanisms, however, need to be clarified in future.

In the current study, we identified 36 upregulated and 3 downregulated genes characterized as LD-associated factors in pancreatic cancer (Table 2). We further identified that enhanced expression of 7 genes namely *CAV2*, *CIDEC*, *HILPDA*, *HSD17B11*, *NCEH1*, *RAB5A*, and *SQLE* are associated with significantly shorter overall survival whereas elevated expression of *BSCL2* and *FITM1* correlates with longer overall survival of pancreatic cancer patients (Fig. 3a). To verify those findings also on the protein level, normal pancreatic tissue samples, in addition to cancerous and para-cancerous tissues, would be required. However, we confirmed protein expression of the DEGs by using the Human Protein Atlas database. Although gene transcripts levels and protein concentrations do not always correspond to each other, we identified prognostic LD-associated factors at the transcriptome level, providing clues for future proteomics, metabolomics and downstream functional analysis.

CAV-2 is a caveolin family member and CAV-2 expression is increased during the accumulation of intracellular LDs and the adipogenic differentiation [43]. CAV-2 plays a key role in intracellular cell transport and higher level of CAV-2 is associated with different types of cancer progression [44]. It has been demonstrated that higher expression of CAV-2 and its upstream regulator bromodomain containing 4 (BRD4) is associated with shorter overall survival of 76 pancreatic cancer patients [44]. In our study, both *CAV1* and *CAV2* were identified as DEGs, but only *CAV2* was a prognostic DEG associated with shorter OS in pancreatic cancer patients.

CIDEC (also known as fat-specific protein of 27 kDa, FSP27) has been suggested to mediate LD-LD contact for promoting LD fusion [6, 10]. CIDE proteins are enriched at LD-LD contact sites and physically chaining the adjacent organelles [10]. So far it has been demonstrated that CIDEC promotes development of hepatic steatosis and steatohepatitis [45, 46]. In our study, only *CIDEC* but not *CIDEA* or *CIDEB* was identified as a prognostic DEG. The precise and specific role of CIDEC in cancer needs to be clarified.

In various cancer cells including renal cell carcinoma, ovarian clear cell carcinoma, colorectal adenoma and carcinoma, upregulation of HILPDA has been observed [47–49]. Hypoxia-inducible factor 1 (HIF1) regulates the expression of HILPDA [11]. HILPDA preferentially accumulates in LDs undergoing remodeling (e.g. expansion). HILPDA has been shown to co-localize with the lipogenic enzymes DGAT1 and DGAT2 [11], which were both identified as DEGs in our study (Table 2). DGATs catalyze the final rate-limiting step in the formation of triglycerides. In line with this, HILPDA has been shown to promote intracellular lipid accumulation by enhancing triglyceride synthesis [11]. Overexpression of HILPDA, but not DGAT1/2, is associated with shorter overall survival in pancreatic cancer patients (Fig. 3a), suggesting that additional roles of HILPDA, other than regulating triacylglyceride (TAG) synthesis, may influence the outcome of pancreatic cancer patients. Indeed, HILPDA inhibits the rate-limiting enzyme of TAG hydrolysis PNPLA2 (ATGL), leading to inhibition of lipolysis, attenuated fatty acid oxidation and ROS production [11].

HSD17B11 is known to convert 5α-androstan-3α, 17β-diol (3α-diol) to androsterone [50]. HSD17B11 regulates size of LDs, LD distribution and TAG content [51]. The role of HSD17B11 in pancreatic cancer has not been elucidated. It has been shown that *HSD17B13* variants are associated with nonalcoholic fatty liver disease (NAFLD) and HSD17B13 expression is elevated in nonalcoholic steatohepatitis (NASH) patients, or with risk of cirrhosis and hepatocellular carcinoma (HCC), while a *HSD17B13* variant has been demonstrated to protect from HCC development [52–54]. In our study, *HSD17B13* was not identified as DEG. Regarding NCEH1, which hydrolyzes 2-acetyl monoalkylglycerol in the metabolism of ether lipids, it has been suggested as a prognostic marker for pancreatic cancer [55], supporting our analysis.

Rab family protein RAB5A belongs to the Ras family of G-proteins, which regulate membrane vesicle trafficking. In our study, several Rab family members namely *RAB5A*, *RAB7A*, *RAB8A*, and *RAB18* were identified as DEGs (Table 2). Among these four genes, expression of *RAB5A* was associated with shorter OS of pancreatic cancer patients. Expression of RAB5A correlated with pancreatic tumor progression in another study of 111 patients as well [56]. Increased expression of RAB5A predicts metastasis and shorter OS in colorectal cancer patients [57]. It has been suggested that RAB5A regulates Wnt signaling, proliferation, invasion and 5-FU drug resistance [56]. Further, RAB5A activates IRS1, Akt and mTOR signaling [58], suggesting that LD-associated factors are also involved in regulating signaling pathways. SQLE is a key enzyme in the cholesterol synthesis pathway and converts squalene to 2,3-epoxysqualene [59]. SQLE increase epigenetic silencing of *PTEN*, leading to activation of the Akt-mTOR pathway and NAFLD-induced HCC growth. High expression of SQLE was associated with shorter OS of HCC patients [60]. On the other hand, it has been demonstrated that reduction of SQLE mRNA and protein expression is associated with shortened survival of colorectal cancer patients. SQLE reduction aggravates colorectal cancer progression via the activation of the β-catenin pathway and deactivation of p53 pathway [61]. In our study with pancreatic cancer patient databases, *SQLE* was identified as a DEG and higher expression of *SQLE* was associated with shorter OS of pancreatic cancer patients. The precise and cancer type-specific role of SQLE has to be further clarified.

### Study strengths and limitations

Bioinformatics analysis revealed that expression of LD-associated factors is associated with overall survival in pancreatic cancer patients. Although aberrant lipid synthesis and reprogrammed lipid metabolism are both associated with the development and progression of pancreatic cancer, LD and LD-associated factors have not been considered in this disease. In the current study, we identified 65 factors as LD-associated factors. We identified 39 DEGs with 36 up-regulated and 3 down-regulated genes. Among 39 DEGs, 7 up-regulated genes and 2 down-regulated genes were significantly associated with overall survival of pancreatic cancer patients. Cox regression analysis further validated 8 factors as prognostic factors.

There are also several limitations of the study. Cofounding factors such as body-mass index (BMI) and others could not be considered, since the data of normal tissue from the GTEx database do not include these information. Furthermore, findings were not validated on the protein level. Further studies must include functional analysis using knockout animal model of the prognostic candidate genes to analyze whether deletion/ inhibition of the lipid droplet-associated factors can change the metabolic profile of the cells, proliferation, and the ability to metastasize. It would also be of interest to clarify whether lipid droplets can be detected in liquid biopsies (blood), and whether deletion/ inhibition of LD-associated factors reduce lipid droplet contents in the blood.

## Conclusions

LDs are ubiquitous cellular organelles, involved not only in lipid metabolism but also in diverse biological functions such as regulating signaling pathways. LDs mediate proliferation, invasion, metastasis, as well as chemotherapy resistance in several types of cancer. LD-associated proteins play an important role in dynamics of LD and it is now evident that expression of several LD-associated genes are associated with overall survival in pancreatic cancer patients. The current study identified prognostic LD-associated factors at the transcriptome level, providing clues for future proteomics and downstream functional and pathway analysis. It is important to increase our understanding of cancer type-specific roles of LD-associated factors, which may help to develop more specific and personalized therapies for pancreatic cancer patients in the future.

## Data Availability

All data generated or analyzed during this study are included in this article.

## Abbreviations

ABHD: Abhydrolase
ACAT: Acetyl-CoA acetyltransferase
ACSL: Acyl-CoA synthetase long chain family member
ADRP: Adipocyte differentiation-related protein
AGPAT: 1-Acylglycerol-3-phosphate O-acyltransferase
ALDI: Associated with LD protein I
ANGPTL: Angiopoietin like
APO: Apolipoprotein
ATGL: Adipose triglyceride lipase
AUP: Ancient ubiquitous protein
BSCL: Berardinelli-seip congenital lipodystrophy
CAV: Caveolin
CAVIN: Caveolae-associated protein
CCT: CTP:phosphocholine cytidyltransferase
CES: Carboxyesterase
CGI: Comparative gene identification
CI: Confidence interval
CIDE: Cell death-inducing DNA fragmentation factor-like effector
DEG: Differentially expressed gene
DFF: Death-inducing DNA fragmentation factor
DGAT: Diacylglycerol O-acyltransferase
ER: Endoplasmic reticulum
FAF: Fas-associated factor
FITM: Fat storage inducing transmembrane protein
FSP: Fat-specific protein
G0S2: G0/G1 switch protein
GEPIA: Gene expression profiling interactive analysis
GPAT: Glycerol-3-phosphate acyltransferase
HCC: Hepatocellular carcinoma
HIF: Hypoxia-inducible factor
HILPDA: Hypoxia inducible LD associated
HR: Hazard ratio
HSD: Hydroxysteroid
HSL: Hormone sensitive lipase
ICE: Interactor of little elongation complex ELL
LD: Lipid droplet
LDAH: LD-associated hydrolase
LIPE: Lipase E
LPCAT: Lysophosphatidylcholine acyltransferase
LPIN: Lipin
LSDP: Lipid storage droplet protein
LSS: Lanosterol synthase
METTL: Methyltransferase like
MGLL: Monoglyceride lipase
MLDP: Myocardial LD protein
MTTP: Microsomal triglyceride transfer protein
NAFLD: Nonalcoholic fatty liver disease
NAP: *N*-ethylmaleimide-sensitive factor attachment protein
NASH: Nonalcoholic steatohepatitis
NCEH: Neutral cholesterol ester hydrolase
NSF: *N*-ethylmaleimide-sensitive factor
OSBPL: Oxysterol binding protein like
ORP: Oxysterol-binding protein-related protein
PAP: Phosphatidic acid phosphohydrolase
PAT: Perilipin, ADRP, TIP47
PCYT: Phosphate cytidylyl transferase
PEMT: Phosphatidylethanolamine *N*-methytransferase
PLD: Phospholipase
PLIN: Perilipin
PNPLA: Patatin-like phospholipase
PTRF: Polymerase I and transcript release factor
SNAP: Synaptosome associated protein
SQLE: Squalene epoxidase
STX: Syntaxin
TIP: Tail-interacting protein
UBE: Ubiquitin conjugating enzyme
UBXD: UBX domain
VAMP: Vesicle associated membrane protein
VAPA: Vesicle-associated membrane protein-associated protein
VCP: Valosin containing protein
VMP: Vesicular membrane protein
